# Protective Effect of Nasal Colonisation with *∆cps/piaA* and *∆cps/proABC* *Streptococcus pneumoniae* Strains against Recolonisation and Invasive Infection

**DOI:** 10.3390/vaccines9030261

**Published:** 2021-03-15

**Authors:** Elisa Ramos-Sevillano, Giuseppe Ercoli, José Afonso Guerra-Assunção, Philip Felgner, Rafael Ramiro de Assis, Rie Nakajima, David Goldblatt, Kevin Kweku Adjei Tetteh, Robert Simon Heyderman, Stephen Brian Gordon, Daniela Mulari Ferreria, Jeremy Stuart Brown

**Affiliations:** 1 Centre for Inflammation and Tissue Repair, UCL Respiratory, Division of Medicine, University College London, Rayne Institute, London WC1E 6JF, UK; g.ercoli@ucl.ac.uk; 2 Great Ormond Street Institute of Child Health, University College London (UCL), London WC1N 1EH, UK; a.guerra@ucl.ac.uk; 3 Vaccine Research and Development Center, Department of Physiology and Biophysics, University of California Irvine, Irvine, CA 92697-4560, USA; pfelgner@hs.uci.edu (P.F.); rafael.assis@outlook.com (R.R.d.A.); rie3@hs.uci.edu (R.N.); 4 Immunobiology Section, UCL Great Ormond Street Institute of Child Health, NIHR Biomedical Research Centre, London WC1N 1EH, UK; d.goldblatt@ucl.ac.uk; 5 Faculty of Infectious and Tropical Diseases, London School of Tropical Medicine and Hygiene, London WC1E 7HT, UK; Kevin.Tetteh@lshtm.ac.uk; 6 Research Department of Infection, Division of Infection and Immunity, University College London, Rayne Institute, London WC1E 6JF, UK; r.heyderman@ucl.ac.uk; 7 Malawi-Liverpool-Wellcome Trust Clinical Research Programme, Blantyre 30096, Malawi; sgordon@mlw.mw; 8 Department of Clinical Sciences, Liverpool School of Tropical Medicine, Liverpool L3 5QA, UK; Daniela.Ferreira@lstmed.ac.uk

**Keywords:** *Streptococcus pneumoniae*, capsule, vaccine, *psaA*, *proABC*, colonisation, immunity

## Abstract

Rationale: Nasopharyngeal administration of live virulence-attenuated *Streptococcus pneumoniae* strains is a potential novel preventative strategy. One target for creating reduced virulence *S. pneumoniae* strains is the capsule, but loss of the capsule reduces the duration of *S. pneumoniae* colonisation in mice which could impair protective efficacy against subsequent infection. Objectives: To assess protective efficacy of nasopharyngeal administration of unencapsulated *S. pneumoniae* strains in murine infection models. Methods: Strains containing *cps* locus deletions combined with the *S. pneumoniae* virulence factors *psaA* (reduces colonisation) or *proABC* (no effect on colonisation) were constructed and their virulence phenotypes and ability to prevent recolonisation or invasive infection assessed using mouse infection models. Serological responses to colonisation were compared between strains using ELISAs, immunoblots and 254 *S. pneumoniae* protein antigen array. Measurements and Main Results: The *∆cps/piaA* and *∆cps/proABC* strains were strongly attenuated in virulence in both invasive infection models and had a reduced ability to colonise the nasopharynx. ELISAs, immunoblots and protein arrays showed colonisation with either strain stimulated weaker serological responses than the wild type strain. Mice previously colonised with these strains were protected against septicaemic pneumonia but, unlike mice colonised with the wild type strain, not against *S. pneumoniae* recolonisation. Conclusions: Colonisation with the *∆cps/piaA* and *∆cps/proABC* strains prevented subsequent septicaemia, but in contrast, to published data for encapsulated double mutant strains they did not prevent recolonisation with *S. pneumoniae*. These data suggest targeting the *cps* locus is a less effective option for creating live attenuated strains that prevent *S. pneumoniae* infections.

## 1. Introduction

*Streptococcus pneumoniae* is the dominant bacterial pathogen causing acute lung infections in adults, responsible for up to 40 to 50% of community acquired pneumonia [1,2] and 25% of exacerbations of COPD [3]. However, existing *S. pneumoniae* vaccines have significant drawbacks. The pneumococcal polysaccharide vaccine (PPV) used in adult risk groups protects against invasive infection (septicaemia) but only has a limited efficacy for preventing *S. pneumoniae* lung infections [4]. The conjugated polysaccharide vaccine (PCV) used routinely prevents pneumonia in children and also in adults [5], as well as herd immunity due to preventing nasopharyngeal colonisation in children [6]. However, PCV vaccination is expensive and only protects against the limited number of capsular serotypes contained within the vaccine [5,7]. As a high proportion of adult disease is caused by non-vaccine serotypes [5] and the prevalence of these is increasing due to serotype replacement in response to vaccination of infants [8,9], PCV vaccination of adults is not cost-effective [10] and the benefits of herd immunity are being lost [7]. New strategies for the prevention of *S. pneumoniae* infections are required that possess broad serotype coverage and are able to induce pulmonary mucosal as well as systemic immunity.

A high proportion of adults have effective naturally acquired immunity against *S. pneumoniae* [11,12], which extensive human and mouse data suggest develops in response to natural *S. pneumoniae* colonisation of the nasopharynx. Colonisation induces antibody and CD4+ Th17 responses to protein antigens and antibody to capsular antigen, preventing recolonisation of the nasopharynx, pneumonia and sepsis [13,14,15,16,17,18,19,20,21,22,23,24]. These responses are likely to contribute to the decrease in rates of *S. pneumoniae* in older children and adults compared to infants [12]. Importantly, data obtained using experimental human colonisation with *S. pneumoniae* have demonstrated that adaptive immunity to *S. pneumoniae* is boosted by recolonisation events [24,25]. Hence, a potential strategy for preventing *S. pneumoniae* infections in adults would be deliberate nasopharyngeal administration of immunizing *S. pneumoniae* strains genetically modified to have markedly reduced virulence. However, to be successful this preventative approach requires live *S. pneumoniae* strains that cannot cause serious infections in susceptible subjects but are still able to colonise the nasopharynx and effectively boost adaptive immunity [13,16].

Recently, we have published pre-clinical data confirming the potential of this approach using two encapsulated mutant strains containing deletions of two loci encoding protein virulence factors (*∆fhs/piaA* or *∆proABC/piaA*) [23]. In murine models both strains colonised the nasopharynx at a similar density at seven days as the wild type strains. Double mutant strains were used to prevent a single reversion event restoring full virulence of the strain, essential for maintaining safety for strains that may be deliberately administered to humans. Previous publications have shown prior exposure to other attenuated strains also induce protective immunity [15,26,27,28]. A major determinant of *S. pneumoniae* virulence is the capsule and unencapsulated mutants very rarely cause invasive disease. Hence, preventing capsule expression could be a particularly safe strategy for making virulence attenuated strains able to stimulate protective immunity. However, we have previously shown that in mice colonisation-induced immunity to *S. pneumoniae* is dependent on the duration of colonisation [13,14,26,29]. As unencapsulated strains have a shorter duration of colonisation of the nasopharynx in mice these data suggest loss of the capsule could have a negative effect on colonisation-induced protection against subsequent *S. pneumoniae* infection.

Here, we describe the design of genetically engineered *S. pneumoniae* strains carrying a deletion of the *cps* locus and genes encoding one of two other protein virulence factors, PsaA [30,31] or ProABC [23,32]. To clarify the utility of targeting the capsule locus for making live attenuated *S. pneumoniae* vaccine strains, the immune response to nasopharyngeal administration of these strains and their protective efficacy against pneumonia with septicaemia and colonisation were tested in mouse models of infection.

## 2. Methods

### 2.1. Bacterial Methods and Construction of the Deletion Mutant Strains

*S. pneumoniae* strains were cultured at 37 °C and 5% CO_2_ on either solid Columbia agar supplemented with 5% horse blood (SLS) or in Todd–Hewitt broth supplemented with 0.5% yeast extract (THY). Spectinomycin (150 µg mL^−1^) and kanamycin (250 µg mL^−1^) were added to blood agar plates when needed. Growth of strains was measured in THY broth by measuring OD_595_ at regular intervals of 0.5 h using a TECAN Spark^®^ plate reader. Working stocks of bacterial cultures in THY (OD_580_ 0.4–0.5) were stored at −80 °C with 10% glycerol. All mutant strains were constructed in the BHN418 capsular serotype 6B clinical *S. pneumoniae* isolate using overlap extension PCR as previously described [29] and the primers shown in Table S1. The constructs were transformed into *S. pneumoniae* by homologous recombination and allelic replacement using competence stimulating peptides (CSP-1, CSP-2) and previously described standard protocols [33].

### 2.2. C3b Complement Binding Assays

Complement factor C3b deposition on *S. pneumoniae* was assessed using an established flow cytometry assay after incubation in human serum. C3b was measured using a fluorescein isothiocyanate (FITC)-conjugated polyclonal anti-human C3 antibody (ICN-Cappel, Canada) [34]. Pooled serum from unvaccinated human volunteers stored as single-use aliquots at −70 °C was used as the source of complement.

### 2.3. Immunofluorescence Microscopy

The reactivity of the anti-pneumococcal antiserum (Statens Serum Institute, Denmark) against pneumococci was determined using a fluorescence-based antibody protocol [35]. Briefly, fresh bacterial cultures grown at an OD_595_ of 0.2–0.3 were incubated with a 1/500 dilution of the serotype 6 antiserum for 30 min and this was followed by incubation with a 1/500 dilution of an anti-rabbit Alexa Fluor 546 antibody (Abcam, UK). A 1/10,000 dilution of DAPI (Biolegend, San Diego, CA, USA) was used as counterstains in order visualize DNA. Samples were examined using a compact confocal laser scanning microscope Zeiss LSM 800.

### 2.4. Immunological Assays

Immunoblots of *S. pneumoniae* lysates and whole cell ELISAs were performed as previously described [13,36]. Briefly, immunoblotting was performed using serum from *S. pneumoniae* colonised mice (1:200), which was detected with a 1/10,000 dilution of goat anti-mouse IgG-HRP (Abcam, UK), following probing of 10 µL aliquots of concentrated bacterial lysates from three different *S. pneumoniae* strains (6B, D39 and TIGR4), prepared from normalized cultures grown to OD_595_ 0.3 and repetitive cycles of boiling and freezing. Lysates were probed with sera obtained day 28 after two episodes of colonisation with 6B, *∆cps/psaA*, *∆cps/proABC,* or sham colonised in order to detect IgG responses. Whole cell ELISAs were performed as previously described [13,36] incubating plates with dilutions of mouse serum for 1  h at room temperature and using HRP-conjugated goat anti mouse IgG or goat anti-mouse IgA (Abcam, UK), incubation with 50 µL TMB chromogen solution (Thermo Fisher Scientific, Waltham, MA, USA) and measuring absorbance at 450 nm using a VersaMax microplate reader.

### 2.5. Protein Microarray Assays

The protein microarray contained 254 proteins selected based on conservation from an original panel of >600 *S. pneumoniae* strains [23,37,38]. Exons were amplified from genomic DNA (strain TIGR4) and cloned into a T7 expression vector. Proteins were expressed incubating the plasmids for 16h in an *E. coli*-based in vitro transcription/translation (IVTT) reaction (Thermofisher, Waltham, MA, USA). Proteins were tested for expression by Western blot using antibodies against N-terminal poly-histidine (His) and printed onto nitrocellulose coated glass AVID slides (Grace Bio-Labs, Bend, OR, USA) using an Omni Grid 100 microarray printer (Genomic Solutions, Ann Arbor, MI, USA). Arrays were probed with mouse serum samples diluted 1:25 in protein array blocking buffer (Maine Manufacturing, Sanford, ME, USA) and supplemented with *E. coli* lysate. Images were acquired and analysed using an ArrayCAM^®^ Imaging System from Grace Bio-Labs.

### 2.6. Mouse Experimental Models

Animal procedures were approved by the local ethical review process and conducted in accordance with the UK national guidelines for animal use and care under project license (PPL70/6510). Pneumonia, sepsis and colonisation models were performed as previously described [11,13,20,22,36] using group sizes of 5+ 4–8 weeks old CD1 mice and inocula sizes for each infection model of: pneumonia, intranasal inhalation 10^7^ CFU in 50 µL PBS; sepsis, intraperitoneal injection of 5 × 10^6^ CFU in 100 µL PBS; colonisation, intranasal inhalation of 10^7^ CFU in 10 µL PBS. To obtain target organ CFU mice were sacrificed after 24–28 h (pneumonia/ sepsis models) or 7 days (colonisation model) using a lethal dose of pentobarbital. For the protection studies mice were colonised on day 0 and day 14 before serum collection or challenge with wild type *S. pneumoniae* on day 28+.

### 2.7. RNA Samples and Sequencing

RNA for RNA-seq were extracted from double mutant strains cultured to an OD_600_ 0.4–0.5 using Mirvana RNA kit (Applied biosystems, Foster City, CA, USA) with an additional physical lysis step using 0.1 mm glass beads (MP Biomedicals, Irvine, CA, USA), treated with Turbo DNase (Applied biosystems, Foster City, CA, USA) and deleted of ribosomal RNA using Ribo-Zero Magnetic Kit Bacteria (Illumina, San Diego, CA, USA) before preparation of sequencing libraries using the KAPA RNA HyperPrep kit (Roche Diagnostics, Basel, Switzerland) and 8 cycles of amplification. Libraries were multiplexed to 24 samples per run and single-end sequenced with the NextSeq 500 desktop sequencer (Illumina, San Diego, CA, USA) using a 75 cycle high-output kit. The RNA sequencing data was mapped and quantified to the *S. pneumoniae* transcriptome 6B BHN418 reference using the Salmon algorithm. Downstream analyses were performed within the R statistical computing framework. The data were integrated into a matrix of raw counts using the TXimport package. The data were then normalised using the DEseq2 package using the rlog method which was used for differential gene expression. This analysis used a p-value cut-off of 0.01 and a cut-off of 1.5 log-fold-change to be considered significantly differentially expressed.

### 2.8. Statistical Analyses

Animal data are presented as median of log_10_ CFU/mL (IQR) recovered from target organs after infection with wild type, *∆cps/psaA*, *∆cps/proABC* or PBS controls. *p*-Values (* < 0.05, ** < 0.01, *** < 0.001) were obtained using Kruskal–Wallis tests with Dunn’s post hoc test comparing groups to the wild type 6B strain (for virulence models), the PBS sham-colonised (for colonisation then challenge data). Quantitative results are expressed as means ± S.D or median with interquartile range for animal experiments. Statistical analyses were performed using GraphPad Prism 8 (GraphPad Software, La Jolla, CA, USA). *p*-Values < 0.05 (95% confidence) were considered statistically significant.

## 3. Results

### 3.1. Design and Characterisation of Unencapsulated Mutant Strains

**Figure 1:**
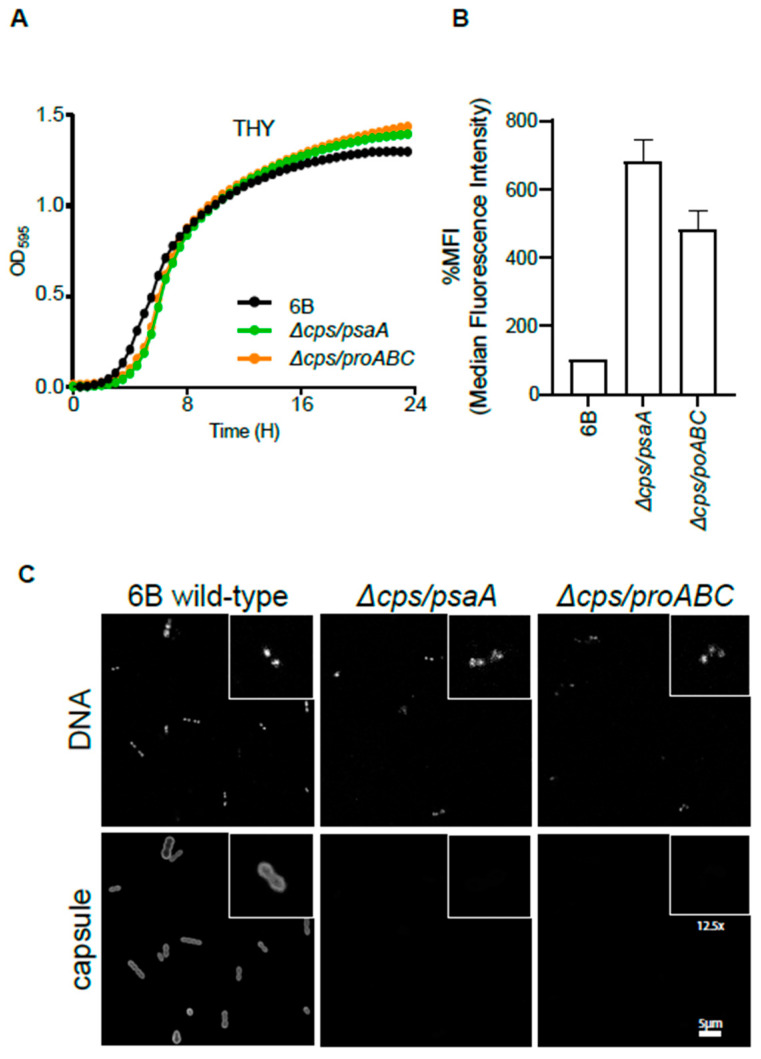
Phenotypic characterization of double unencapsulated mutants ∆*cps/psaA* and ∆*cps/proABC*. (**A**) Growth of wild type and unencapsulated strains *∆cps/psaA* and *∆cps/proABC* in THY assessed by measuring OD595 for a 24 h period. (**B**) Effect of the *S. pneumoniae* capsule on C3b deposition. Median fluorescence intensity (MFI) of C3b deposition measured using flow cytometry on the wild type 6B and unencapsulated mutants when incubated in 25% human serum. Error bars represent SDs and asterisks represent the differences between wild type and the unencapsulated mutant strain. For both mutant strains, *p* is < 0.0001 (one way ANOVA) compared to the wild type strain. (**C**) Fluorescent microscopy of wild type and unencapsulated double mutant strains following incubation with 4′,6-diamidino-2-phenylindole (DAPI) (binds to DNA to identify bacterial cells, top panels) or pneumococcal antiserum labelled with Alexa fluor 546 (recognizes serotype 6 capsule, bottom panels). The scale bar (bottom right) represents 5 µm and the inserts show a 12.5 higher magnification of selected bacteria.

To test the potential efficacy of unencapsulated *S. pneumoniae* strains for preventing subsequent infections, a mutant strain containing complete deletion of the *cps* locus was constructed in the BHN418 capsular serotype 6B strain background. This strain was selected as it has low virulence in humans and is used in the Liverpool Experimental Human Pneumococcal Carriage model (EHPC) [24]. To minimise the chance of revertants leading to recovery of virulence when used in human studies, double mutant strains were created combining deletion of the *cps* locus with a deletion of either the *psaA* [39] or *proABC* [23,32] virulence genes to make the unencapsulated *∆cps/psaA* and *∆cps/proABC* strains. Loss of *psaA* is known to reduce murine nasopharyngeal colonisation, whereas loss of *proABC* has no effect [23,32]. Both mutants were stable after multiple rounds of growth without antibiotic selective pressure (data not shown) and showed no growth defect in complete media compared to the wild type strain (Figure 1A). As expected from previous publications, both unencapsulated strains were considerably more sensitive to complement recognition than the wild type parental strain (Figure 1B). Loss of the capsule in both strains was also confirmed by immunofluorescence (Figure 1C).

### 3.2. Virulence of ∆cps/psaA and ∆cps/proABC Strains

**Figure 2:**
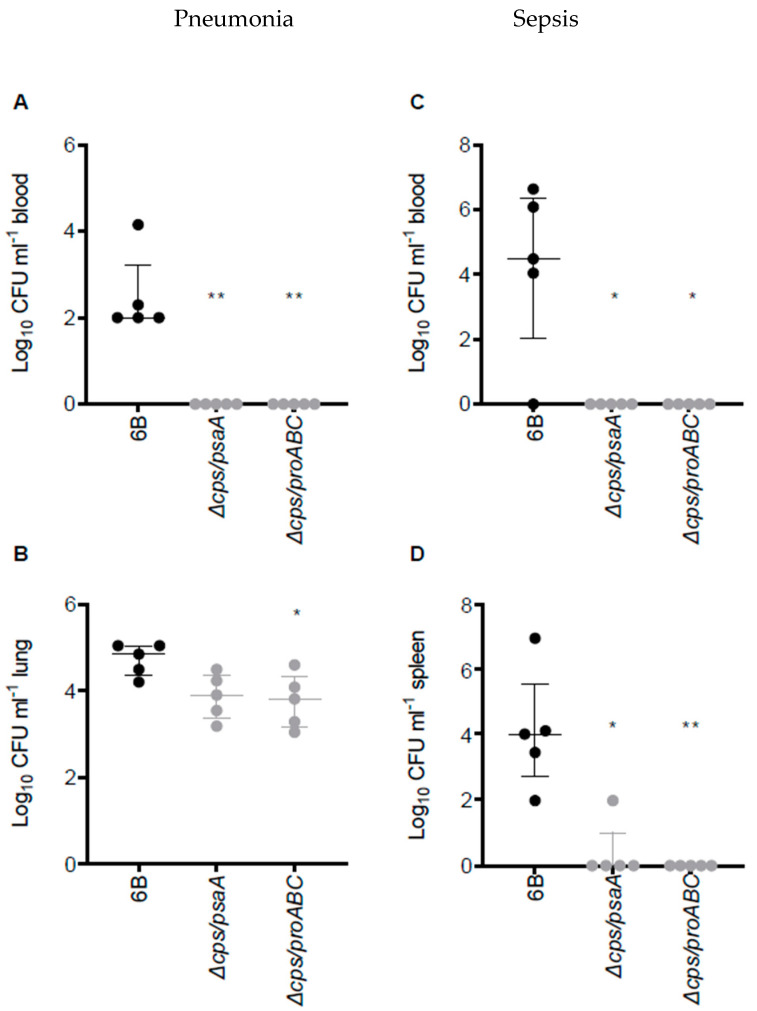
Virulence of double unencapsulated mutant strains in murine pneumonia and sepsis models. (**A**,**B**) Pneumonia model; CFU obtained from blood (**A**) and lung (**B**) of CD1 mice 28 h post intranasal inoculation with 1 × 10^7^ CFU of wild type 6B or double mutant *S. pneumoniae* strains. (**C**) and (**D**) Sepsis model; CFU in blood (**C**) or spleen (**D**) of CD1 mice 24 h post intraperitoneal inoculation with 5 × 10^6^ CFU of wild type 6B or double mutant *S. pneumoniae* strains. Each symbol represents CFU data from a single mouse (black symbols, wild type *S. pneumoniae* infection, grey mutant *S. pneumoniae* strain infection), horizontal bars represent median values, error bars represent interquartile range and asterisks represent statistical significance compared to the wild type strain (Kruskal–Wallis with Dunn’s post hoc test to identify significant differences between groups, * *p* < 0.05; ** *p* < 0.01).

The virulence phenotype of ∆*cps/psaA* and ∆*cps/proABC* strains were assessed in an established CD1 mouse model of pneumonia and septicaemia (Figure 2). As expected, for both mutant strains almost no colony-forming units (CFU) were recovered in either model from blood or spleen, indicating that bacteria were unable to spread from lungs to blood or rapidly cleared when they reached the blood. Both the ∆*cps/psaA* and *∆cps/proABC* mutant strains had lower lung CFU than the wild type 6B strain but this was only statistically significant for the *∆cps/proABC* mutant (Figure 2B).

### 3.3. RNA-seq Analysis of the Unencapsulated Double Mutant Strains

**Figure 3:**
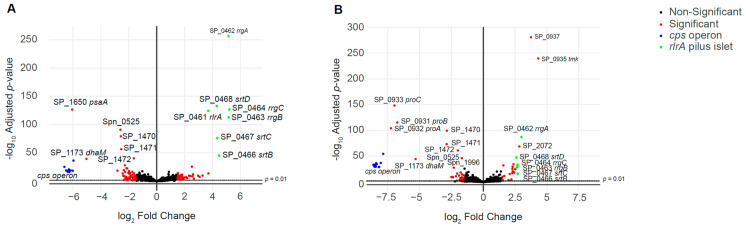
Volcano plots showing differential expression of genes in the live attenuated strains. Log_2_ ratio gene expression levels for the mutant strains (**A**) *∆cps/psaA* and (**B**) *∆cps/proABC* compared to the wild type strain. Fold change expression is represented in the X-axis, with negative values indicating genes that are under-expressed in the mutant versus the wild type, while positive fold change values indicate genes that are over-expressed in the mutant versus the wild type. The Y-axis shows the *p* values (as -log_10_ values) for differentially expressed genes. Genes belonging to *cps* locus are shown in blue, while those belonging to the pilus are shown in green, other genes that are differentially expressed are represented in red and all other genes are represented in black. Differential gene expression and statistical significance were analysed using the DESeq2 method. The dashed line above the *x* axis marks the significance threshold of *p* = 0.01. A full list of the genes showing differential expression with their expression levels is shown in Table S2, with selected genes of interest shown in Table 1.

To ensure there had been no unexpected effects of the mutations that could compromise safety if the double mutant strains were used in the EHPC model, RNA-seq was used to provide an overview of the effects of the mutations on pneumococcal gene expression. For this analysis, RNA was isolated from mid log-phase cultures in THY of the wild type, *∆cps/psaA* and *∆cps/proABC* strains. RNA-seq identified a relatively low number of significantly up- or down-regulation of multiple genes in the double mutant strains (Figure 3, Table S2). Using a log_2_ cut-off of 1.5, 46 and 28 genes were up-regulated in *∆cps/psaA* and *∆cps/proABC,* respectively. Several of the upregulated genes showed increased expression in both mutant strains, suggesting they could be a response to loss of the *cps* locus. Table 1 is showing the most important changes found. These included genes encoding an alcohol dehydrogenase XylB (Spn_00124), the adjacent transcriptional regulator and cation efflux system protein (Spn_00125, Spn_00126), MutT (Spn_00677), AliA (Spn_00914, just downstream of the capsule operon) and interestingly the *rlrA* islet locus which encodes expression of the pilus (Spn_01010-15; *rrgA*, *rrgB*, *rrgC*, *srtB*, *srtC* and *srtD*) (Table 1). Upregulated genes specific to the *∆cps/psaA* strain included those that encoded proteins involved in type II fatty acid biosynthesis (Spn_00963, fabM, a enoyl-CoA hydratase/isomerase; Spn_00967, a trans-2-enoyl-ACP reductase II; and Spn_00968_fabD, a malonyl CoA-acyl carrier protein transacylase), along with the Blp bacteriocin loci (Spn_0599-600, blpUO; and Spn_01098-1100, blpIJK; and Spn_01108_BlpX, Spn_01111) (Table 1). For the ∆*cps/proABC* mutant there was increased expression of an operon predicted to encode a fructose phospostransferase transporter system (PTS) (Spn_02091_tkt, Spn_02092_alsE, Spn_02093_fruA_2, Spn_02094_manP, Spn_02095_hrsA, Spn_02096_ptsN, Spn_02097_licR_2) (Table 1). Importantly, the double mutant strains did not have increased expression of the well-described virulence factors such as Ply, PspA and PspC (Figure 3).

The total number of downregulated genes were 64 and 46 in *∆cps/psaA* and *∆cps/proABC*, respectively. As expected, RNA transcripts from the *cps* locus (both strains), *psaA* (*∆cps/psaA* strain), or *proABC* operon (*∆cps/proABC* strain) were barely detected in RNA from the mutant strains. Other genes showing reduced expression included several genes involved in carbohydrate metabolism, including those encoding a sugar PTS (Spn_00618-621, PTS-EIIB, PTS-EIIC, manZ_2 and PTS_EII_2), GalT and GalK *(Spn_00121-122)* involved in galactose metabolism, the ABC-type glycerol-3-phosphate transport system (Spn_00641-642, the permease and substrate-binding protein of CUT1) [40] and the Lac operon II (Spn_01723-726, *lacGEFT*). Endo-beta-N-acetylglucosaminidase was significantly decreased only in *∆cps/psaA* (Spn_01042-46) and *bgaC* in *∆cps/proABC* (Spn_00617).

### 3.4. Nasopharyngeal Colonisation by Double Mutant Strains and Subsequent Serological Responses

**Figure 4:**
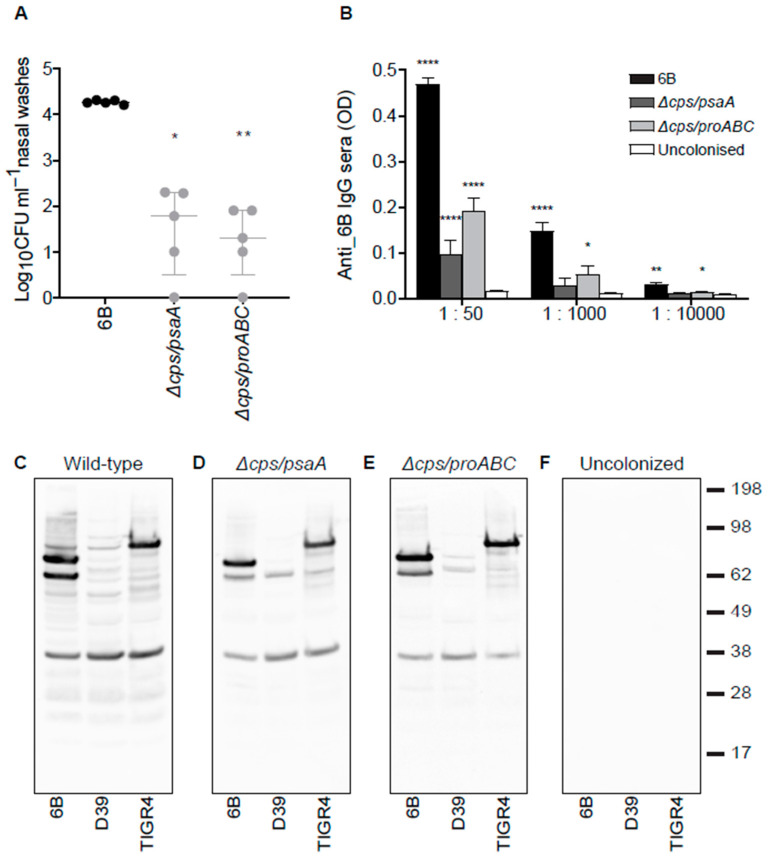
Wild type 6B and the unencapsulated double mutant strains induce a systemic antibody response after nasopharynx colonisation. (**A**) Colonisation model; nasal wash CFU 7 days post colonisation of CD1 mice with 1 × 10^7^ CFU of wild type 6B or the double mutant *S. pneumoniae* strains. (**B**) Whole-cell enzyme-linked immunosorbent assay (ELISA) anti-6B immunoglobulin (Ig)G responses in mouse sera 28 days post-colonisation with the corresponding strain 6B (black bars), *∆cps/psaA* mutant (dark grey bars), *∆cps/proABC* mutant (light grey bars) compared with uncolonised controls (white bars). *N* = 5 for each group and the data analysed using Kruskal-Wallis with Dunn’s post hoc test to identify significant differences between selected groups; *, *p* < 0.05; **, *p* < 0.01 ****, *p* < 0.0001. (**C**–**F**) IgG immunoblots for whole-cell lysates of three different *S. pneumoniae* strains (6B, D39 and TIGR4) probed with sera obtained 28 days after two episodes of colonisation with 6B (**C**), *∆csp/psaA* (**D**), *∆cps/proABC* (**E**) strains, or from PBS sham colonized mice (**F**).

The ability of the ∆*cps/psaA* and ∆*cps/proABC* strains to colonise the nasopharynx was assessed in an established mouse model of colonisation. Both double mutant strains had a significant defect in colonising ability, with nasal wash CFU two log_10_ lower than that for the wild type strain 7 days after inoculation (Figure 4A). Despite this, serum recovered 21 days after two episodes of colonisation with the double mutant strains demonstrated significant serum whole cell ELISA IgG responses to the homologous 6B strain (Figure 4B). These were markedly lower than the ELISA titres induced by colonisation with the wild type 6B *S. pneumoniae* strain, which potentially could reflect loss of anti-capsular IgG responses. Hence, the anti-protein antigen responses were specifically assessed using immunoblots against whole cell lysates of three different strains of *S. pneumoniae*. These confirmed serum IgG from colonised mice recognised multiple protein bands in *S. pneumoniae* 6B, TIGR4 and D39 strains lysates with similar band patterns for different serum samples and different target strains, suggesting the major protein targets were generally conserved (Figure 4C). The immunoblots demonstrated fewer protein bands were recognised by serum from mice colonised with the ∆*cps/psaA* and ∆*cps/proABC* strains compared to serum from mice colonised with the wild type strain. These data suggested there was a weaker serological response to colonisation with the ∆*cps/psaA* and ∆*cps/proABC* strains compared to colonisation with the wild type strain.

### 3.5. Identification of Protein Antigens Recognised by Serological Responses to Colonisation Using Protein Microarrays

**Figure 5:**
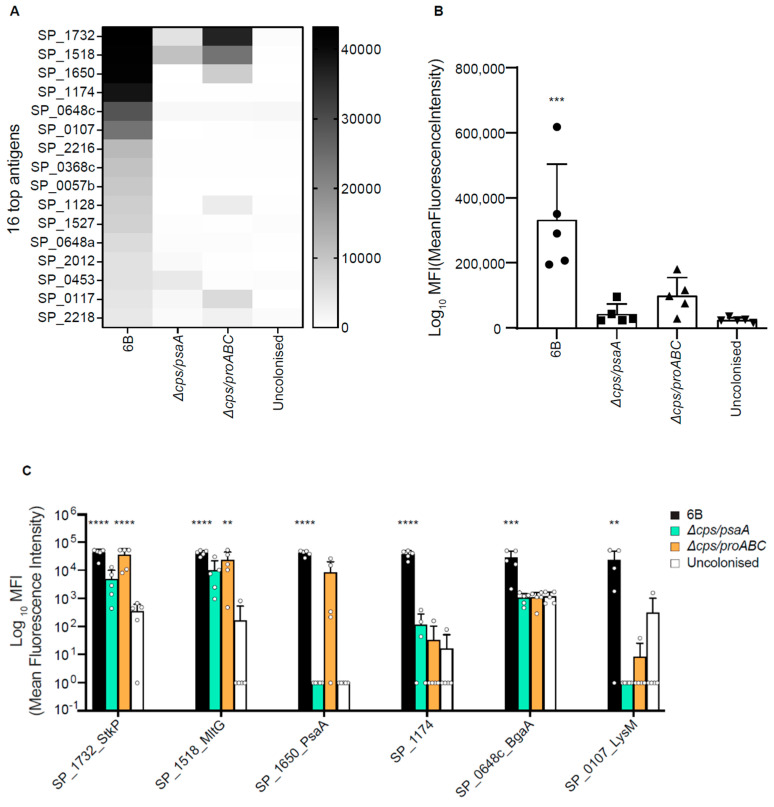
Identification of the protein antigens recognized by IgG in serum from mice colonised with wild type and double mutant *S. pneumoniae* strains. Antigen specific IgG binding data obtained by probing a protein array containing 254 *S. pneumoniae* protein antigens with sera from mice colonised twice with the 6B, *∆cps/psaA*, or *∆cps/proABC* strains. (**A**) Heatmap of mean IgG binding levels to the top 16 proteins recognised by IgG in colonised mouse sera (*n* = 5 mice). (**B**) Aggregated MFI for IgG binding to the 30 antigens with the highest level of IgG binding in serum from mice colonised twice with the 6B, *∆cps/psaA*, or *∆cps/proABC* strains, or sham colonized with PBS. Each symbol represents data from 1 mouse, bars represent mean and error bars SD. Asterisks represent statistical significance compared to the uncolonised group (Kruskal-Wallis with Dunn’s multiple comparisons posthoc test; ***, *p* < 0.001). (**C**) Binding results in sera from mice colonised twice with the 6B strain, *∆cps/psaA*, or *∆cps/proABC* strains, or sham colonised to the 6 antigens with the highest level of IgG binding in serum obtained from wild type colonized mice. Each symbol represents data from 1 mouse, bars represent mean and error bars SD. Asterisks represent statistical significance compared to the uncolonised group (two-way ANOVA with Dunnett’s for multiple comparisons, **, *p* < 0.01; ***, *p* < 0.001, ****, *p* < 0.0001).

A protein microarray containing 254 major conserved *S. pneumoniae* proteins recognised by naturally acquired antibody found in human sera [37,41] was used to identify which protein antigens were recognised by IgG in sera from mice colonised with the wild type, ∆*cps/psaA* or ∆*cps/proABC* strains. The list of protein antigens on the array is reported in the Supplementary materials (Table S3). Comparison of the IgG-specific signal for the top 16 antigens after incubation with sera from colonized mice showed greater IgG response for the group of mice colonised with wild type 6B (Figure 5A). For the pooled data to the top 30 antigens responses for each individual mouse, only the serum from wild type colonised mice showed significantly greater IgG responses to mock colonised mice (*p* < 0.001) (Figure 5B). In addition, comparing the results for the most abundant protein antigens individually, IgG in sera from 6B colonized group showed statistically significant responses to the serine/threonine protease StkP (SP_1732), the transglycosidase MltG (SP_1518), PsaA (SP1650), SP_1174, BgaA (SP_0648) and the LysM domain protein (SP_0107). In contrast, sera from mice colonised with the *∆cps/proABC* mutant showed significant increases in IgG just to StkP (SP_1732) and MltG (SP_1518) and sera from *∆cps/psaA* colonised mice did not show any significant differences to specific antigens compared to sera from the control uncolonised group (Figure 5C). These data confirm that colonisation with the *∆cps/proABC* and particularly the *∆cps/psaA* strain induced weaker anti-protein antigen responses compared to colonisation with the wild type strain.

### 3.6. Double Mutant Colonisation Protects against Bacteraemia during Pneumonia Challenge

**Figure 6:**
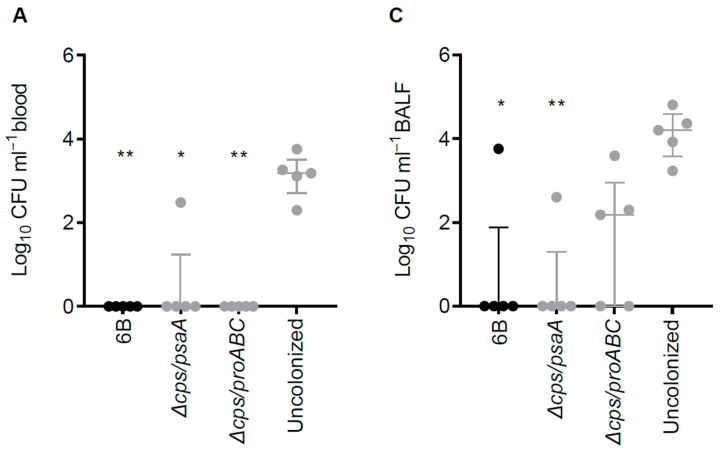

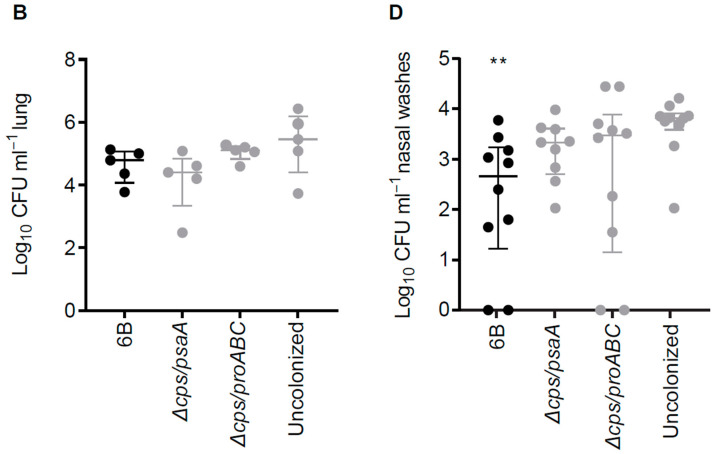
Rechallenge of mice previously colonised with 6B and the double mutant attenuated strains using wild type 6B and pneumonia and colonisation models. (**A**–**C**) Target organ CFU for sham-colonised, 6B colonised, or double mutant strain colonised CD1 mice challenged 30 days after colonisation by intranasal inoculation of 1 × 10^7^ CFU wild type 6B *S. pneumoniae.* (**A**) Blood, (**B**) lung homogenate and (**C**) BALF *S. pneumoniae* CFU (log_10_ ml^−1^) 24 hours following the pneumonia challenge. (**D**) Nasal wash CFU 7 days after intranasal recolonisation challenge of CD1 mice with 1 × 10^7^ CFU of the *S. pneumoniae* 6B strain 42 days after two episodes of colonisation with the wild type 6B or double mutant strains. Each symbol represents data from a single mouse, horizontal bars represent median values, error bars represent interquartile range and asterisks represent statistical significance compared to sham colonised group (Kruskall–Wallis with Dunn’s post hoc test; *, *p* < 0.05; **, *p* < 0.01).

The protective efficacy of prior colonisation with the unencapsulated double mutant strains was assessed by two colonisation episodes followed by challenge with the 6B wild type strain 30 days later using pneumonia and colonisation models. Mice previously colonised twice with either wild type or the double mutant pneumococcal strains were totally protected against septicaemia (Figure 6A) after wild type *S. pneumoniae* challenge. In contrast, mice colonised with the wild type, *∆cps/psaA* and *∆cps/proABC* strains did not show a significant reduction of bacterial CFU within the lung after pneumonia challenge (Figure 6B), although the number of CFU recovered from BALF was significantly reduced after colonisation with the *∆cps/psaA* mutant compared to uncolonised group (Figure 6C). Protection against nasopharyngeal colonisation challenge with the wild type 6B was assessed by using nasal wash CFU 7 days after inoculation. Prior colonisation with the wild type strains reduced nasal wash CFU by about one log_10_ compared to sham colonised control mice when recolonised with the 6B strain (Figure 6D). However, neither the *∆cps/psaA* or *∆cps/proABC* strains reduced nasal wash CFU compared to sham colonised control mice, showing that in contrast to the data for double mutant encapsulated virulence attenuated strains [23] previous colonisation with unencapsulated strains had no protective efficacy against subsequent recolonisation.

## 4. Discussion

*S. pneumoniae* nasopharyngeal colonisation is an immunising event and almost all adult humans have important levels of protective immunity against *S. pneumoniae* [42,43,44,45]. Deliberately administering immunizing *S. pneumoniae* to the nasopharynx could boost existing adaptive immune mechanisms that prevent nasopharyngeal colonisation (the precursor for all invasive infections), lung infection and septicaemia [20,24] as well as alveolar macrophage mediated innate immunity [46,47]. We have recently published pre-clinical data showing the feasibility of this approach using double mutant encapsulated *S. pneumoniae* strains [23] and have now extended these data to evaluate the protective efficacy of unencapsulated double mutant strains. The results show that both of our unencapsulated strains were highly attenuated in virulence, although unlike the double mutant strains targeting protein virulence determinants [23], they also had an impaired ability to colonise the nasopharynx. Although colonisation with either mutant stimulated an antibody response this was weaker than the response to colonisation with the wild type strain. Furthermore, although mice previously colonized with either mutant were protected against pneumonia with septicaemia, they were not protected against recolonisation with *S. pneumoniae*.

The *S. pneumoniae* capsule inhibits complement mediated opsonophagocytosis and is largely essential for systemic virulence of *S. pneumoniae* [34,39], so is an attractive target for making attenuated *S. pneumoniae* strains for use as vaccines. As any attenuated strains intended for use in humans will need to contain mutations of two separate virulence determinants to ensure safety, deletions of the *cps* locus were combined with deletion of either *psaA*, a previously described virulence gene that encodes the lipoprotein component of the dominant *S. pneumoniae* manganese uptake ABC transporter [48], or *proABC*, required for proline biosynthesis and recently shown to be important for virulence [23,49]. As the *∆proABC* does not affect colonisation [23] and we have previously linked loss of the *cps* locus to reduced colonisation [13,14,22], the reduced nasopharyngeal colonisation with the *∆cps/proABC* strain was likely to be due to loss of the capsule. RNA-seq showed limited changes in gene transcription between the *∆cps/psaA*, *∆cps/proABC* and wild type strains suggesting these mutations were unlikely to have caused unexpected effects that might compromise their safe use. The exception was that loss of the *cps* locus was associated with increase expression of the genome islet that encodes the *S. pneumoniae* pilus which might be predicted to increase adhesion of *S. pneumoniae* to epithelial surfaces [50,51]. However, the reduced levels of colonisation with the unencapsulated strains indicate the increased expression of the pilus islet had little functional significance.

Whole cell ELISA data and immunoblots against bacterial lysates suggested that colonisation with wild type *S. pneumoniae* induced stronger antibody responses to protein antigens than colonisation with the unencapsulated mutant strains. These data were confirmed by the results of probing the *S. pneumoniae* protein antigen array, which demonstrated that colonisation with the *∆cps/psaA* or *∆cps/proABC* strains induced statistically significant increases in IgG to none or only two protein antigens, respectively. In contrast, colonisation with the wild-type strain induced IgG to six protein antigens including well recognised protective antigens such as SktP and PsaA. Although the protein array contains only 254 of the most immunogenic protein antigens in human data [37,41] and therefore will miss responses to several other protein antigens not included on the array, these results collectively demonstrate there is a weaker adaptive immune response to the *∆cps/psaA* and *∆cps/proABC* strains. This could be the case because the mutation has reduced antigen expression, with the most obvious example being PsaA the gene for which is deleted in the *∆cps/psaA* mutant. However, of the 6 antigens recognized after colonisation with the wild type strain, only *SP_0648* and *psaA* showed significant reduced expression by the *∆cps/psaA* mutant strain (log_2_ fold change, −1.694 and −6.048 respectively) and none for the *∆cps/proABC* strain. The most likely reason for the overall reduced immune response to the *∆cps/psaA* and *∆cps/proABC* strains is their reduced density of colonisation of the nasopharynx, as our previous data have shown that a lower level of nasopharyngeal colonisation results in a weaker immune response [13,14].

Despite the reduced immune responses to nasal administration of the double unencapsulated mutant strains, this still provided strong protection against subsequent septicaemia caused by pneumonia re-challenge. Presumably, even the weaker antibody response to protein antigens induced by colonisation with the *∆cps/psaA* and *∆cps/proABC* strains is adequate to control septicaemia; another possibility is that T cell mediated immunity (not assessed here) was unaffected by the capsule mutation. However, the latter seems less likely as in mouse models prevention of recolonisation is thought to largely depend on T cell mediated immunity [21] and colonisation with the *∆cps/psaA* and *∆cps/proABC* strains did not prevent recolonisation with wild type *S. pneumoniae* unlike colonisation with the wild type or encapsulated mutant strains (as shown by our previous data) [23]. Overall, these results combined with our previous observations [13,22] suggest that although a lower level of colonisation with mutant *S. pneumoniae* strains can induce enough anti-protein antibody to prevent septicaemia, prevention of recolonisation needs a higher level of immunity induced by a more sustained initial colonisation of the nasopharynx by the attenuated strains. Whether this is due to reduced antibody responses in nasal wash or sera after colonisation with the unencapsulated strains, or potential effects on the strength of cellular responses will require further investigation. Other limitations of this study include only assessing antibody responses to 254 protein antigens which is likely to have missed important antibody responses and lack of data on CD4 cellular responses or on protection against pneumonia caused by a heterologous strain.

## 5. Conclusions

To summarise, we have previously shown that colonisation of the nasopharynx with mutant attenuated strains could be a cost-effective strategy to support current vaccination programmes that can overcome some of the limitations of vaccines based on capsular antigens [23]. Nasal administration of live attenuated *S. pneumoniae* strains could improve prevention of pneumonia in adults with considerable benefits for morbidity and mortality. However, here we demonstrate that although colonisation with unencapsulated strains can prevent septicaemia, it does not prevent subsequent recolonisation with wild type *S. pneumoniae* and therefore is likely to have limited effects on immunity to *S. pneumoniae* in the respiratory tract. These data provide further information on the parameters affecting the success or otherwise of a strategy using virulence attenuated *S. pneumoniae* strains to prevent future infections and suggest targeting the *cps* locus is a less attractive approach for this strategy.

## Figures and Tables

**Table 1:** RNA-seq data. Selected *S. pneumoniae* genes and operons showing statistically significant differential expression between the double mutant strains and the wild type 6B strain when cultured to mid-log growth phase in THY broth. Gene numbers for both BHN418 and the TIGR4 strain are given, along with gene names where these have been described. Data are presented as log_2_ fold change (either for a single gene or mean and SD values for all genes in an operon) and include only genes with >1.5 log_2_ differences. Genes or operons marked in bold are deleted in the mutant strains (*cps* locus, Spn_00899-913; *psaA*, Spn_02120; *proABC*, Spn_01479-81).

BHN418 Gene Number and Name or Operon Function	TIGR4 Gene Number (If Known)	*∆cps/psaA*	*∆cps/proABC*
***Upregulated genes***			
Spn_00124_*xylB*	SP_1855	2.956	1.538
Spn_00125_6 regulator/cation efflux	SP_1856-7	3.493 (0.293)	2.099 (0.159
Spn_00599-00 Blp bacteriocin	SP_0041-	1.838 (0.127)	
Spn_00677_*mutT*	SP_0119	1.533	1.502
Spn_00914_*aliA*	SP_0366	2.520	2.677
Spn_00963-68 fatty acid synthesis	SP_0415-419-420	1.623 (0.153)	
Spn_01010-15_*rlrA islet*	SP_0462-68	4.766 (0.438)	2.706 (0.157)
Spn_01098-00 Blp bacteriocin	SP_0531-33	2.168 (0.510)	
Spn_01108_*blpX*	SP_0544	2.561	
Spn_01111 Blp bacteriocin	SP_0547	1.506	
Spn_02091-97 fructose PTS	SP_1615-21		2.354 (0.083)
***Downregulated genes***			
Spn_00121-22 Galactose metabolism	SP_1852-53	−2.058 (0.134)	−1.690 (0.006)
Spn_00617_*bgaC*	SP_0060		−1.698
Spn_00618-21 sugar PTS	SP_0060-64	−2.158 (0.238)	−2.273 (0.317)
Spn_00641-42 glycerol ABC transporter	SP_0091-92	−2.344 (0.125)	−1.669 (0.183)
**Spn_00899-913 *cps* locus**	**SP_0343-65**	**−6.304 (0.164)**	**−8.385 (0.220)**
Spn_01042-46 Endo-beta-N-acetylglucosaminidase	SP_0498	−1.771 (0.331)	
**Spn_01479-81_*proBAC***	**SP_0931-33**		**−6.992 (0.247)**
Spn_01723-26_*lacGEFT*	SP_1184-87	−1.978 (0.281)	−1.831 (0.023)
**Spn_02120_*psaA***	**SP_1650**	**−6.048**	

## Data Availability

Raw RNA-seq data have been deposited in the ArrayExpress database at EMBL-EBI (www.ebi.ac.uk/arrayexpress, accessed on 14 March 2021) under accession number E-MTAB-10076.

## Supplementary Materials

The following are available online at https://www.mdpi.com/2076-393X/9/3/261/s1, Table S1: Pneumococcal strain, plasmids and primers used in this study. Table S2: RNA-seq data. Table S3: Protein array whole dataset.
