# Supplementary material for: Protective Effect of Nasal Colonisation with ∆cps/piaA and ∆cps/proABC Streptococcus pneumoniae Strains against Recolonisation and Invasive Infection
Source: Vaccines (Basel). 2021 Mar 15;9(3):261. doi: 10.3390/vaccines9030261 (PMC8000150; doi:10.3390/vaccines9030261)
Supplement: Supplementary file 1 [file vaccines-09-00261-s001.zip › Suppl Material/Suppl Table 1.docx]

| **Strain** | Primer name | Primer sequence | Description |
| --- | --- | --- | --- |
| 6B(BHN418) |  |  | *S. pneumoniae* capsular serotype 6 |
| psaA | PsaA_UpF  PsaA_UpspecF  PsaA_UpspecR  PsaA_DownspecF  PsaA_Downspec R  PsaA_DownR | ggaggtgacctatgattgc  gccctaataaattggaggatctagatcccccgtttgattttt  aaaatcaaacgggggatctagatcctccaatttattagggct  aaattggatccattccgcgtcgcctctgaaaaacgtcattctc  atgacgtttttcagaggcgacgcggaatggatcca  atattatccacgtattcaacgtagcga |  |
| spr832 | Spr832_UpF  Spr832_UpspecF  Spr832_UpspecR  Spr832_DownspecF  Spr832_Downspec R  Spr832_DownR | ccaaacgggtatcttgttacag  tgttattcatgttataatggagatcccccgtttgattt  atccattaaaaatcaaacgggggatctccattataacatgaat  aaaaattggatccattccgcgtcagctttgactgcctctttt  aaagaggcagtcaaagctgacgcggaatggatccaat  ggaaactaccaatgctgtcttgttt |  |
| cps | Cps_UpF  Cps_UpKanF  Cps_UpKanR  Cps_DownKanF  Cps_DownKanR  Cps_DownR | ggattgataaaggtattggtggt  gctttctgtgtggaattactataaatattgtcgatactatgttatacgccaac  gttggcgtataacatagtatcgacaatatttatagtaattccacacaga  cttttctgaagtacatccgcaacgaaaatgatgaaaagttcaaaac  gttttgaacttttcatcattttcgttgcggatgtacttcagaaaag  cagtttgtccattcaactgag |  |
| **Plasmids** |  |  |  |
| pR412 |  |  | Derived from ColE1, carrying a 1145 bp minitransposon that contains Himar1 IRs flanking add9 gene. SpcR (*Martin B et al., 2000*) |
| pABG5mini |  |  | Derived from pMGC66, an E. coli-streptococcal shuttle vector that harbors phoZF (*Granok AB et al., 2000*) |
