# Supplementary material for: Protective Effect of Nasal Colonisation with ∆cps/piaA and ∆cps/proABC Streptococcus pneumoniae Strains against Recolonisation and Invasive Infection
Source: Vaccines (Basel). 2021 Mar 15;9(3):261. doi: 10.3390/vaccines9030261 (PMC8000150; doi:10.3390/vaccines9030261)
Supplement: Supplementary file 1 [file vaccines-09-00261-s001.zip › Suppl Material/Suppl Table 2.docx]

**Supp Table 2. RNA-seq data.** *S. pneumoniae* genes showing statistically significant differential expression between the double mutant strains and the wild type 6B strain when cultured to mid-log growth phase in THY broth. Gene numbers for both BHN418 and the TIGR4 strain are given, along with gene names where these have been described. Data are presented as log_2_ fold change, and include only genes with >1.5 log_2_ differences. Genes marked in bold are deleted in the mutant strains (*cps* locus, Spn_00899-913; *psaA*, Spn_02120; *proABC*, Spn_01479-81).

| **BHN418 gene number and name** | **TIGR4 gene number**  **(if known)** | ***∆cps/psaA*** | ***∆cps/proABC*** |
| --- | --- | --- | --- |
| ***Upregulated genes*** |  |  |  |
| Spn_00044_queF | SP_1777 |  | 1.576 |
| Spn_00045_aqpZ2 | SP_1778 | 1.524 |  |
| Spn_00124_xylB | SP_1855 | 2.956 | 1.538 |
| Spn_00125_adhR | SP_1856 | 3.285 | 1.986 |
| Spn_00126_czcD | SP_1857 | 3.700 | 2.211 |
| Spn_00169_IS1167 |  | 1.615 |  |
| Spn_00274 | SP_1992 | 1.827 | 2.081 |
| Spn_00287 | SP_2004 | 1.627 |  |
| Spn_00382_guaA_1 | SP_2072 |  | 2.827 |
| Spn_00434 | SP_2125 | 1.601 |  |
| Spn_00577_IS1167 |  | 2.098 |  |
| Spn_00578 | SP_2170 | 3.066 |  |
| Spn_00580_tadA | SP_0020 | 1.610 |  |
| Spn_00599_blpU | SP_0041 | 1.748 |  |
| Spn_00600_blpO_2 |  | 1.928 |  |
| Spn_00677 | SP_0119 | 1.533 | 1.502 |
| Spn_00683_cibA | SP_0125 |  | 1.655 |
| Spn_00699 | SP_0142 | 1.515 |  |
| Spn_00716 | SP_0159 | 2.400 |  |
| Spn_00814_sorC | SP_0247 | 1.523 |  |
| Spn_00851_pbuO | SP_0287 | 1.513 |  |
| Spn_00852 | SP_0288 | 1.536 |  |
| Spn_00914_aliA | SP_0366 | 2.520 | 2.677 |
| Spn_00963_fabM | SP_0415 | 1.554 |  |
| Spn_00967 | SP_0419 | 1.799 |  |
| Spn_00968_fabD | SP_0420 | 1.517 |  |
| Spn_00996 | SP_0449 | 1.963 |  |
| Spn_01009 | SP_0461 rlrA | 3.686 |  |
| Spn_01010 | SP_0462 rrgA | 5.138 | 3.000 |
| Spn_01011 | SP_0463 rrgB | 5.156 | 2.693 |
| Spn_01012 | Sp_0464 rrgC | 5.194 | 2.689 |
| Spn_01013 | Sp_0466 srtB | 4.470 | 2.701 |
| Spn_01014 | Sp_0467 srtC | 4.343 | 2.545 |
| Spn_01015 | Sp_0468 srtD | 4.297 | 2.606 |
| Spn_01032_cspR | SP_0488 | 1.761 |  |
| Spn_01098_blpI | SP_0531 | 1.650 |  |
| Spn_01099 | SP_0532 | 2.184 |  |
| Spn_01100_blpN_1 | SP_0533 | 2.669 |  |
| Spn_01108_blpX | SP_0544 | 2.561 |  |
| Spn_01111 | SP_0547 | 1.506 |  |
| Spn_01482_tmk | SP_0935 |  | 4.321 |
| Spn_01483_holB | SP_0936 |  | 3.978 |
| Spn_01484 | SP_0937 |  | 3.748 |
| Spn_01485_rsmI | SP_0938 |  | 3.650 |
| Spn_01501_pyrDII | SP_0963 |  | 2.259 |
| Spn_01565_yfhA | SP_1034 | 1.541 |  |
| Spn_01567 | SP_1582 | 1.845 | 1.514 |
| Spn_01810_pyrR | SP_1278 | 1.501 |  |
| Spn_01819_pyrP | SP_1286 | 1.818 |  |
| Spn_01842 |  | 1.525 |  |
| Spn_02062_cshA_2 | SP_1586 | 1.747 |  |
| Spn_02087 | SP_1611 | 2.318 |  |
| Spn_02088 | SP_1612 | 2.603 |  |
| Spn_02091_tkt | SP_1615 |  | 2.374 |
| Spn_02092_alsE | SP_1616 |  | 2.304 |
| Spn_02093_fruA_2 | SP_1617 |  | 2.491 |
| Spn_02094_manP | SP_1618 |  | 2.278 |
| Spn_02095_hrsA | SP_1619 |  | 2.437 |
| Spn_02096_ptsN | SP_1620 |  | 2.279 |
| Spn_02097_licR_2 | SP_1621 |  | 2.317 |
| ***Downregulated genes*** |  |  |  |
| Spn_00015 | SP_0498 | -1.813 |  |
| Spn_00063_sacA | SP_1795 | -1.741 |  |
| Spn_00120_paaI | SP_1851 | -1.710 |  |
| Spn_00121_galT | SP_1852 | -2.153 | -1.686 |
| Spn_00122_galK | SP_1853 | -1.963 | -1.694 |
| Spn_00278 | SP_1996 | -2.115 | -2.319 |
| Spn_00358 | SP_2063 | -1.614 |  |
| Spn_00452_bglK_1 | SP_2142 | -1.624 |  |
| Spn_00453_mngB | SP_2143 | -1.515 |  |
| Spn_00494_glpO | SP_2185 | -1.531 |  |
| Spn_00525 |  | -2.596 | -1.684 |
| Spn_00615_strH | SP_0057 | -1.568 |  |
| Spn_00617_bgaC | SP_0060 |  | -1.698 |
| Spn_00618_PTS-EIIB | SP_0060 | -1.826 | -1.806 |
| Spn_00619_PTS-EIIC | SP_0062 | -2.390 | -2.387 |
| Spn_00620_manZ_2 | SP_0062 | -2.189 | -2.382 |
| Spn_00621_PTS-EII_2 | SP_0064 | -2.225 | -2.515 |
| Spn_00641_ycjP_2 | SP_0091 | -2.255 | -1.539 |
| Spn_00642 | SP_0092 | -2.432 | -1.798 |
| Spn_00644 | SP_0096 | -1.949 | -1.902 |
| Spn_00645 | SP_0097 | -1.707 |  |
| Spn_00803_cysW |  | -2.272 | -1.779 |
| Spn_00804 |  | -1.903 |  |
| Spn_00805_potA_1 | SP_0242 | -2.156 | -1.556 |
| Spn_00806 | SP_0243 | -2.349 | -2.055 |
| Spn_00811_yehU_1 | SP_0155 | -1.985 | -1.679 |
| **Spn_00899_wzg** | SP_0346 | **-6.376** | **-8.636** |
| **Spn_00900_cpsB** | SP_0347 | **-6.295** | **-8.473** |
| **Spn_00901_cap5A** | SP_0348 | **-6.364** | **-8.547** |
| **Spn_00902_wze** | SP_0349 | **-6.349** | **-8.478** |
| **Spn_00903_wcaJ_2** |  | **-6.479** | **-8.607** |
| **Spn_00904_gspA_1** |  | **-6.342** | **-8.208** |
| **Spn_00905** |  | **-6.286** | **-8.416** |
| **Spn_00906_kfoC_3** |  | **-6.256** | **-8.443** |
| **Spn_00907** |  | **-6.165** | **-8.367** |
| **Spn_00908** |  | **-6.005** | **-8.425** |
| **Spn_00909_cps2L** |  | **-6.355** | **-8.499** |
| **Spn_00910_rfbC** |  | **-6.372** | **-8.179** |
| **Spn_00911_rmlB** |  | **-6.365** | **-8.584** |
| **Spn_00912_rmlD** |  | **-6.602** | **-8.055** |
| **Spn_00913** |  | **-5.954** | **-7.857** |
| Spn_00983_gutB |  | -2.808 | -2.148 |
| Spn_01007_pfl | SP_0459 | -1.544 |  |
| Spn_01042 | SP_0498 | -1.507 |  |
| Spn_01043 | SP_0498 | -1.566 |  |
| Spn_01044 | SP_0498 | -1.577 |  |
| Spn_01045 | SP_0498 | -1.916 |  |
| Spn_01046 | SP_0498 | -2.288 |  |
| Spn_01053 | SP_0498 | -1.853 |  |
| Spn_01195_gatC_2 | SP_0647 | -1.555 | -1.559 |
| Spn_01196 |  | -2.015 |  |
| Spn_01197_lacZ | SP_0648 | -1.694 |  |
| Spn_01232 | Sp_0684 | 1.608 |  |
| Spn_01252 | SP_0715 | -1.701 |  |
| Spn_01427 | SP_0879 | -1.516 |  |
| **Spn_01479_proB** | **SP_0931** |  | **-6.754** |
| **Spn_01480_proA** | **SP_0932** |  | **-7.248** |
| **Spn_01481_proC** | **SP_0933** |  | **-6.975** |
| Spn_01636_glgA | SP_1124 | -1.549 |  |
| Spn_01712_dhaM | SP_1173 | -5.024 | -5.317 |
| Spn_01722 | SP_1183 |  | -2.081 |
| Spn_01723_lacG | SP_1184 | -2.238 | -1.830 |
| Spn_01724_lacE_2 | SP_1185 | -2.120 | -1.862 |
| Spn_01725_lacF_2 | SP_1186 | -1.959 | -1.807 |
| Spn_01726_lacT | SP_1187 | -1.593 | -1.826 |
| Spn_01732 | SP_1194 |  | -1.579 |
| Spn_01957_apbE | SP_1470 | -2.564 | -2.865 |
| Spn_01958_azr_1 | SP_1471 | -2.535 | -2.877 |
| Spn_01959_azr_2 | SP_1472 | -1.633 | -2.000 |
| **Spn_02120_psaA** | **SP_1650** | **-6.048** |  |
| Spn_02166 |  | -2.027 |  |
